# School eye health – going beyond refractive errors

**Published:** 2015

**Authors:** Sumrana Yasmin, Hasan Minto, Ving Fai Chan

**Affiliations:** Regional Director: Brien Holden Vision Institute, Islamabad, Pakistan. S.Yasmin@brienholdenvision.org; Director: Sustainable Services Development, Brien Holden Vision Institute, Islamabad, Pakistan. h.minto@brienholdenvision.org; Research Manager, Africa: Brien Holden Vision Institute, Durban, South Africa. vingfaic@brienholdenvision.org.za

**Figure:**
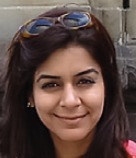
Sumrana Yasmin

**Figure:**
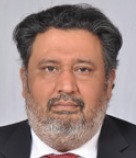
Hasan Minto

**Figure:**
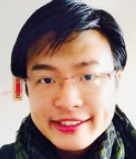
Ving Fai Chan

Health, including visual health, is inextricably linked to school achievement, quality of life, and economic productivity.^1^ Introducing health education in schools is essential as knowledge and good habits acquired at an early age are likely to persist.

Globally, 19 million children are living with vision impairment^2^ and approximately 12 million children have a significant, uncorrected refractive error. Of particular concern is the rapid increase in myopia, particularly in East Asia, where 78% of children in China are affected.^3^

School eye health programmes, when integrated into broader school health education and backed up by eye and child health services, can reach a large number of children and their families.

School eye health can encompass the following:

- **Health promotion and prevention** to increase awareness among children and teachers and to promote a healthy school environment. This can reduce the impact of local endemic eye diseases such as trachoma.
- **Primary eye care** to detect and treat common eye conditions (e.g. infections), refer people with conditions such as cataract, and to manage refractive errors with high quality, appealing and affordable spectacles.

Activities may include:

- Training children to spread eye health messages and conduct simple vision screening among peers and family members (the child-to-child approach).
- Showing children and adults how to help and interact with those who are blind or have irreversible low vision.

Children should be offered general vision screening when they enter and leave primary school, and when they leave secondary school/high school. Any child with visible eye conditions (squint, white pupil, red eyes) and associated symptoms (abnormal head/face turn, inability to copy from the blackboard, complaints of chronic headaches), should also be screened and provided with, or referred to, the appropriate services.

**Figure:**
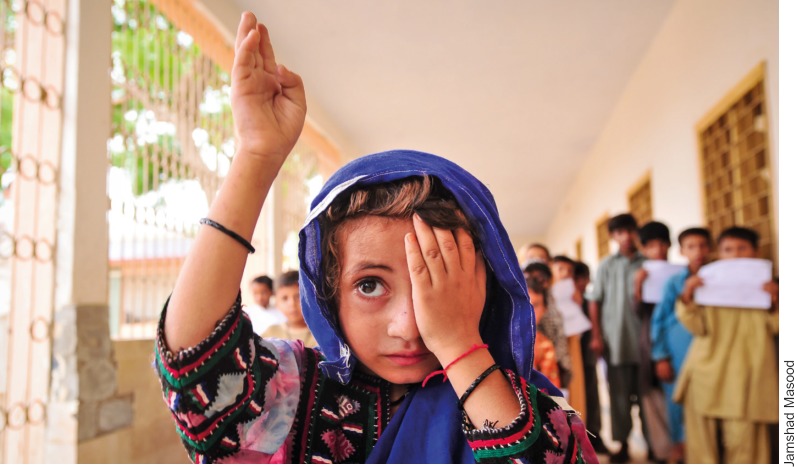
Visual health is linked to school achievement. PAKISTAN

The ideal is to conduct eye health screening for children and teachers in school, and refer those who need further management to the eye unit for examination, refraction and dispensing of spectacles. Another option is to screen and refract the children in the school and allow them to choose a frame they like. The local eye unit can cut lenses, fit them and deliver the spectacles to the school.

Factors that contribute to a successful school eye health programme include:

- The support and engagement of the local education authorities.
- The involvement of parents/carers.
- The enforcement of policies and guidelines to prevent unnecessary prescribing (see below).
- Financial support for optical correction from the government (child health services/insurance schemes).
- Qualified personnel to fit affordable and good quality spectacles.

Spectacles should **not** be prescribed to children with minimal refractive error. Children will not notice a significant improvement in their vision and will therefore simply not wear them! This is a waste of resources.

The guidelines for correction are:

- myopia ≥−0.50D
- hypermetropia ≥+2.00D
- astigmatism ≥0.75D

To increase follow-up and referral, the following must be systematically recorded.

- Uptake of referrals (to ensure services are accessed, including low vision care).
- Spectacle wearing after 3–4 months and any reasons for non-wear.
- Any educational adjustments made for children identified with irreversible vision impairment (by consulting with teachers).
- New and/or progressed myopia cases and replacement of broken/missing spectacles (by repeating screening of 11–15 year-old children).

In order to increase coverage, members of school health programmes can work with school nurses and teachers after consultation with educational authorities.

In order to make informed decisions, research (which can be multi-disciplinary) plays a pivotal role in providing evidence, which might be needed for:

- Planning – needs assessment based on prevalence data, reviews of existing resources and analysis of policy.
- Improving implementation – operational research to identify gaps and challenges could improve the efficiency, effectiveness and quality of programmes.
- Assessing impact – in terms of satisfaction, academic achievement, quality of life, etc.

Eye health is an essential part of a school health programme and should be comprehensive and respond to the locally relevant eye conditions and diseases. Correction of refractive errors is critical but should not be the only focus of a school eye health programme.

**Figure 1.**
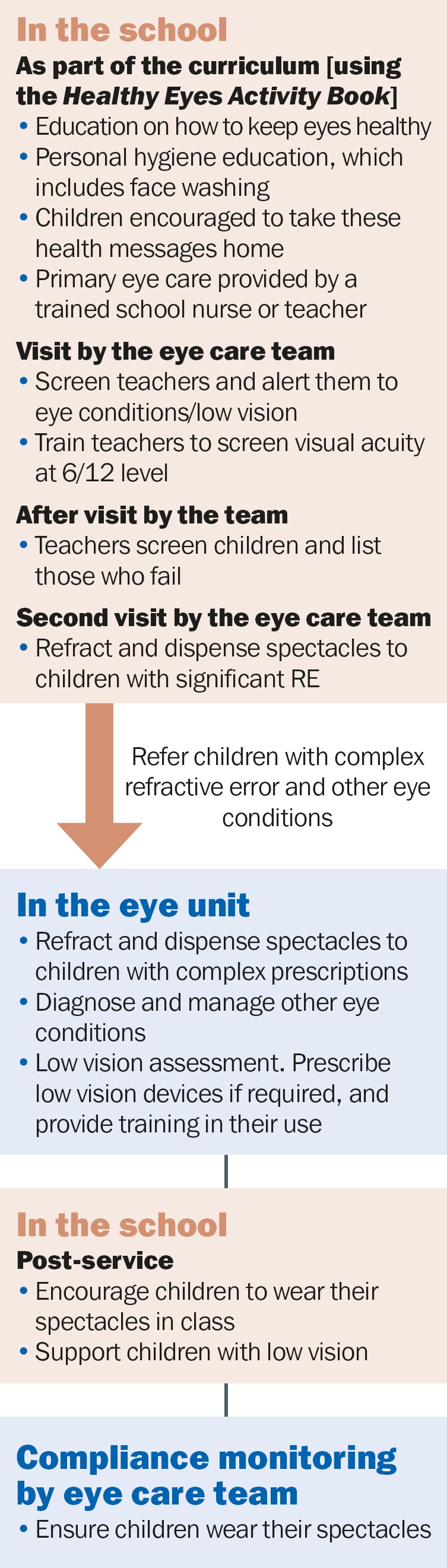
A systematic approach to school eye health

Figure 1 describes a systematic approach to school eye health.
